# Noninvasive Cardiac Electrical Activity Mapping Systems: Current Available Options

**DOI:** 10.31083/RCM44335

**Published:** 2025-11-21

**Authors:** Andrea Ballatore, Andrea Saglietto, Elena Cavallone, Gaetano Maria De Ferrari, Matteo Anselmino, Veronica Dusi

**Affiliations:** ^1^Division of Cardiology, Cardiovascular and Thoracic Department, “Città della Salute e della Scienza” Hospital, 10126 Turin, Italy; ^2^Department of Medical Sciences, University of Turin, 10124 Turin, Italy

**Keywords:** ventricular tachycardia, noninvasive mapping, ECGi, stereotactic radiation ablation, STAR

## Abstract

Electrocardiographic imaging (ECGi) is an innovative noninvasive mapping technique. Indeed, ECGi enables the identification of the earliest points of cardiac activation in both atrial and ventricular focal arrhythmias, as well as rotors and high-frequency domains that could act as potential drivers of atrial fibrillation. Currently, ECGi is most widely used in the management of ventricular tachycardia (VT). Meanwhile, in cases of macro-reentrant arrhythmias, ECGi assists in outlining the re-entry circuit and identifying the myocardial exit site. Additionally, current research is focusing on detecting myocardial scars and critical isthmuses. This information is particularly valuable for planning stereotactic arrhythmia radioablation procedures for VT in patients where invasive electroanatomic maps are unavailable, and a fully noninvasive approach is preferred. The present review aims to examine commercially available options for noninvasive ECG mapping (Amycard, CardioInsight, VIVO, Acorys, and vMAP), highlighting key features and limitations.

## 1. Introduction

Since the first electrocardiographic recording of human cardiac activity, significant advancements have been made in electrocardiogram (ECG) interpretation. However, the underlying principles and technology of the ECG have remained largely unchanged. In recent decades, alongside the traditional ECG used in clinical practice, a growing area of research has focused on solving the so called “inverse problem” of electrocardiography and, subsequently, introducing noninvasive mapping of cardiac electrical activity [1]. Noninvasive ECG mapping is in fact an attractive option to identify the site of origin of arrhythmias, especially in case of frail patients, in whom a full noninvasive diagnostic and therapeutic protocol may be preferable [2, 3]. Noninvasive ECG mapping can be used to identify the origin site of ventricular premature depolarizations (VPDs) and focal tachycardias, and, in macro-reentrant arrhythmias, can delineate the reentry circuit and pinpoint the myocardial exit site of the tachycardia.

Aim of the present review is to describe the clinical scenarios in which noninvasive ECG mapping has been tested and the characteristics of commercially available systems.

## 2. Noninvasive Cardiac Electrical Activity Mapping, the Inverse Problem of Electrocardiography

**Fig. 1.**
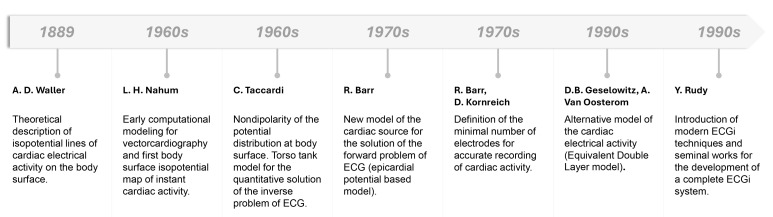
**Timeline of the principal development in ECGi history**. ECGi, electrocardiographic imaging.

The core challenge of electrocardiographic imaging (ECGi) lies in solving the inverse problem of electrocardiography and, eventually, enabling reliable body surface mapping (BSM) (Fig. 1). Although a comprehensive discussion of this topic is beyond the scope of this review, understanding some basic concepts is essential to appreciate the limitations of current BSM systems.

The inverse problem of electrocardiography can be defined as the reconstruction of the heart’s electrical activity based on the processing of electrical signals recorded from the body surface. In a sense, the clinical interpretation of the ECG represents a pragmatic, human approach to solving this problem. Mathematically, this process presents two key challenges: it is non-unique, as different cardiac configurations could theoretically produce the same body surface signals; and it is ‘ill-posed’, meaning that small changes in the initial conditions (e.g., due to noise) can lead to significant variations in the solution. Therefore, resolving the inverse problem of electrocardiography involves defining mathematical constraints that enable a unique, physiologically plausible solution [4].

### 2.1 Cardiac Models

At the core of constructing an ECGi workflow is the modeling of the heart’s electrical activity. In brief, solving the inverse problem requires the development of a forward mathematical model. Two main approaches can be adopted for this purpose [5]. The first is the extracellular potential-based model, which considers the potential at the cardiac surface (epicardium or both epicardium and endocardium) as the electrical source. This approach has several advantages: the surface potential is directly measurable, providing immediate access to potential and electrogram data, and activation maps can be easily reconstructed. However, it lacks direct information on the transmural propagation of the electrical impulse [6].

The second approach is the myocardial activation time model (such as the equivalent double-layer model), which directly describes local activation timing without requiring potential reconstruction. An advantage of this model is that it can represent transmural propagation of the electrical impulse. Earlier versions lacked information on the repolarization phases [6], but this limit has now been solved [7].

The signals recorded at the body surface are influenced not only by the heart’s electrical activity but also by the conductive properties of surrounding tissues. As a result, ECGi models must also account for the heterogeneous, patient-specific torso conductor, incorporating its varying properties and geometries to accurately reconstruct the cardiac electrical activity [8].

### 2.2 Validation Studies

All cardiac models require validation to confirm reliability and clinical validity. This involves evaluating the technical rigor of the ECGi model and its validity in various clinical scenarios. Technical validation includes simulation and experimental setups. Simulation studies use analytical or numerical approaches: analytical methods, though constrained by simple geometry, reveal the importance of source position and relative insensitivity to thorax conductivity [9, 10]; numerical models are less constrained by geometry and allow the investigation of each element of the model separately, investigating how they singularly affect the solution [11, 12]. Experimental setups usually feature a torso tank with a suspended *ex vivo* animal heart, recording electrical activity from the heart and tank surface, enabling accurate quantitative assessments [13]. Animal models are also used, but lack of precise geometrical information impair quantitative analysis [14, 15]: however, a recent experiment using a computed tomography (CT) scan in anesthetized dogs, showed a 10 mm spatial resolution in identifying pacing beat origin [16]. On the other side, clinical validation assesses ECGi accuracy by comparing it with invasive recordings, testing the model’s ability to locate electrical impulse origins and analyze depolarization sequences using mapping electrodes [17].

In any case, the comparison between ECGi and invasive mapping systems requires precise anatomical alignments between maps. Even small errors may have a significant impact on the results: the alignment process is challenging, and should be based on clear and fixed anatomical landmarks as the aortic root, the common pulmonary artery and cava veins [18].

## 3. Noninvasive Cardiac Electrical Activity Mapping, Current Clinical Applications and Areas of Research

Currently, ECGi is primarily used in cardiac electrophysiology to study and manage arrhythmias. In atrial arrhythmias, particularly in atrial fibrillation (AF) [19, 20], ECGi holds the potential to identify focal sources, analyze arrhythmogenic substrates, and localize rotors and drivers linked to successful ablation sites. However, due to the extensive nature of the ablation sets, validation studies in this context are challenging. Few studies [21, 22] have investigated ECGi in Wolff-Parkinson-White (WPW) syndrome for accessory pathway localization and preprocedural risk-benefit assessment, but the main indication is to localize the focal origin of premature ventricular depolarizations (VPD) and ventricular tachycardia (VT). Though results are still suboptimal, ECGi is also being explored for substrate mapping to identify myocardial scars, and, in case of macro-reentrant ventricular arrhythmias, to highlight reentry circuits and critical isthmuses [23] (currently investigated in the ECGI-VT study NCT03713866). While invasive electroanatomical 3D mapping remains the gold standard in these scenarios, ECGi may aid in cases of fast, poorly tolerated or unmappable VTs.

### Stereotactic Radiation Ablation of Ventricular Tachycardia

Catheter ablation is an effective treatment for VT and is increasingly recommended [24]. Its success depends on the underlying cardiac condition, achieving the best outcomes for monomorphic VTs of ischemic etiology. Traditional electroanatomical 3D mapping of the VT circuit requires reproducible induction and hemodynamic tolerance, possible in only few patients. In addition, even when mapped, ablation may be incomplete if the substrate is intramural, epicardial, or near vital structures. For these reasons, when catheter ablation is unfeasible or ineffective, stereotactic arrhythmia radioablation (STAR) has become a realistic option. STAR delivers high-dose radiation (typically 25 Gy in a single session), completely un-invasively, to the arrhythmogenic substrate inaccessible by catheter [2, 3]. Precise pre-procedural planning is essential using prior ablation data, ECGi, or both, followed by respiratory-gated CT imaging to define the final target volume.

The ENCORE-VT [2, 3] demonstrated STAR’s safety and efficacy, obtaining VT/VPD reduction in 94% of the 19 patients treated. In this study the noncommercially available CADIS-ECGI system [1, 25, 26] developed by the group of Rudy *et al*. was used. This ECGi uses 224–250 body surface electrodes paired with an anatomical CT scan to reconstruct the epicardial surface of both the atrial and ventricular chambers and create voltage, activation, and repolarization maps.

Driven by the first, positive, clinical experiences a first joined European Heart Rhythm Association/Heart Rhythm Society Consensus document on STAR workflow has been released [27] and a European multidisciplinary consortium, the STOPSTORM [28] has gathered forces in the attempt to standardize procedural workflow, follow-up reporting, and description of complications.

To date, STAR represents a reasonable option in at least three clinical conditons: (1) critically ill patients, with a VT burden that significantly affects quality of life, presenting high predicted invasive ablation complication rate or low sedation protocol tolerance; (2) patients in which the critical VT isthmus or focal origin is not reachable due to anatomical or technical limitations (e.g., epicardial adhesions from previous surgery, presence of a left ventricular assist device) or “inaccessible” left ventricle (presence of left ventricular thrombosis; mitral and aortic mechanical valves); (3) cases in which invasive catheter ablation has failed (e.g. target deep within the myocardium or protected by fat or fibrous tissue).

In these scenarios, noninvasive ECGi mapping can be crucial [29]. In particular, deep myocardial VTs, in which the earliest activation area with traditional invasive mapping may appear wider and less contingent, are excellent candidates for STAR. In any case, ECGi’s accuracy is deemed to be adequate for STAR due to its broader lesion coverage, incorporating safety margins (typically at least 5 mm) [30, 31]. Moreover, the information deriving from noninvasive mapping can be merged with clinical and imaging details on anatomy and fibrosis and, recently, a tool (HeaRTmaP) has been developed to integrate this information into a radiation planning system [32].

Though literature supports ECGi in STAR planning [33, 34, 35, 36, 37] its use across STOPSTORM centers was limited up to 2022 [28]. Notably, while the STOPSTORM consortium has reported benchmarks in structure [38] for contouring and radiotherapy planning [39], specific guidance on target definition workflows is still lacking.

Limitations related to the standardized use of ECGi include small validation series, initial focus on idiopathic VTs, and difficulty managing multiple morphologies. Improvements are needed in algorithms for activation sequence mapping, distinguishing epicardial from endocardial arrhythmias and identifying diastolic potentials.

## 4. Commercially Available ECGi Systems

Currently, five ECGi systems are commercially available. Table 1 (Ref. [40]) summarizes their main features. The landmark work by Rudy led to the creation of the first commercially available system, CardioInsight™ (Medtronic), approved in Europe in 2012 and in USA in 2014 [26]. In the following decade, other four systems were introduced. Two—Amycard and Acorys—share the same extracellular potential model, requiring many torso electrodes for noninvasive reconstruction. Amycard is the only system offering both epicardial and endocardial reconstructions based on an activation/recovery times model; CardioInsight and Acorys provide only epicardial maps. The remaining systems use different approaches: VIVO applies an equivalent double layer model based on activation time and vMAP employs artificial intelligence (AI) with a forward solution. Both VIVO and vMAP rely on a standard 12-lead ECG. Notably, only Amycard and vMAP do not require 3D imaging in their workflows.

**Table 1.** **Comparison of the features of the commercially available noninvasive ECG mapping systems**.

	Cardio-Insight	Amycard	Acorys	VIVO	vMAP
ECG leads	252 unipolar body-surface electrodes	224 unipolar body-surface electrodes	128 unipolar body-surface electrodes	12 leads ECG (direct, real time recording but also 12 leads Holter ECG data uploading)	12 leads ECG
Model	Extracellular potential model	Extracellular potential model	Extracellular potential model	Equivalent double layer model (activation time)	AI based system utilizing a forward solution approach
Regulatory status	CE (2012) and FDA (2014) marketing approval	CE marketing approval	CE marketing approval (07/2024)	CE (2018) and FDA (2019) marketing approval	FDA (2021) marketing approval
Surface mapped	Epicardium	Epicardium and endocardium	Epicardium	Epicardium and endocardium	No distinction up to 2022 [40]
Maps	Potential map, activation map propagation map, unipolar voltage map, slew rate map.	Isopotential, isochronal, phase map, voltage maps, activation maps.	Isopotential, isochronal, phase map, voltage maps, activation maps, conduction velocity maps.	Activation map.	Specific for the type of arrhythmias.
3D Imaging	CT	CT or MRI	CT or MRI Not strictly necessary	CT or MRI	Not required
Atrial maps	Yes	Yes	Yes (system focused on AF)	No	Yes
VT mapping accuracy compared to invasive mapping	Median distance between ECGi identified earliest activation times and invasive mapping = 22.6 mm (15.8 mm in NICM vs. 26.6 mm in ICM, *p* = 0.055). Worst results when using the earliest negative voltage.	No direct (in mm) comparison available.	No direct (in mm) comparison available.	No direct (in mm) comparison available.	Median spatial accuracy for VT was reported to be 14 mm with excellent regional localization in patients, 23% of whom had SHD.
		Perfect match (PM = same anatomic segment, in a model with 22 LV and 12 RV segments) in 76%, near match (NM) in 97% (n = 37 procedures).		Perfect match (PM = same anatomic segment, in a model with 22 LV and 12 RV segments) in 95%, near match (NM) in 100% (n = 21 procedures).	
Limits	Inability to reconstruct septal structures.	Lower performance in mapping pacing beats from the septum, apical region, and outflow tract.	Inability to reconstruct septal structures.	Suboptimal resolution of sub-aortic and sub-pulmonary valve sites.	
	Struggles to map the full macro-reentrant circuit and activation sequence of arrhythmias.		Unreliable in distinguishing between epicardial and endocardial origins.		
	Unreliable in distinguishing between epicardial and endocardial origins.				
Main research/clinical applications so far	Ventricular arrhythmias treatment.	VT treatment.	Atrial mapping, particularly for AF mechanistic understanding and treatment planning (mostly rotors and high-frequency drivers mapping).	Ventricular arrhythmias treatment.	Ventricular and atrial arrhythmias treatment.
		Identification of regional delay for CRT planning and optimization.	Identification of VT isthmuses during sinus rhythm through conduction velocity maps (substrate maps).		

Legend: AI, artificial intelligence; CE, Conformité Européenne; CRT, cardiac resynchronization therapy; CT, computed tomography; FDA, U.S. Food and Drug Administration; ICM, ischemic cardiomyopathy; MRI, magnetic resonance imaging; NICM, non ischemic cardiomyopathy; SHD, structural heart disease; VT, ventricular tachycardia; ECG, electrocardiogram.

### 4.1 CardioInsight

**Fig. 2.**
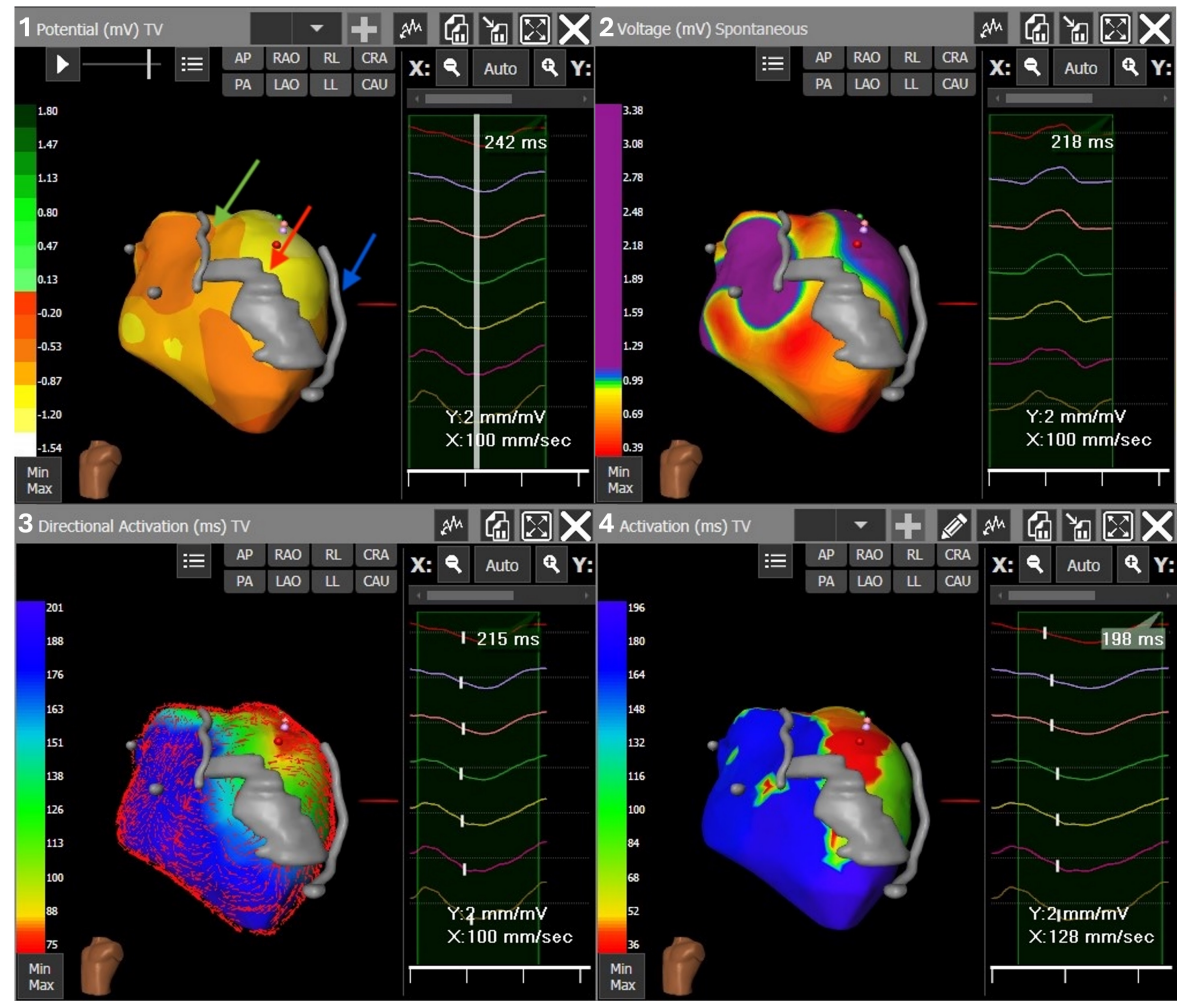
**ECGi mapping performed with the CardioInsight system, LAO projection**. Panels from left to right, up to down: (1) VT potential map providing a snapshot of unipolar activation. In gray the reconstruction of the LAD artery (indicated by the green arrow), a LV medio-apical calcification (indicated by the red arrow) and the LV epicardial catheter (indicated by the blue arrow). (2) Voltage map during atrial pacing showing a medio-apical and inferior scar; the scar is represented by voltages lower than 1 mV in red and non-purple colors, whereas normal voltages are displayed in purple. (3) VT directional activation map showing directional vectors of local activations. (4) VT activation map (first activation in red and latest in blue). The three maps combined suggest a reentry macrocircuit with a large epicardial exit area including the medio-basal antero-lateral LV wall. Legend: LAO, left anterior oblique; LAD, left anterior descending; LV, left ventricle; STAR, stereotactic arrhythmia radioablation; VT, ventricular tachycardia.

CardioInsight (Medtronic, Minneapolis, MN, USA) mapping system reconstructs and maps epicardial potentials exclusively, using the method of fundamental solutions to address the inverse problem of electrocardiography [41]. Both atrial and ventricular chambers can be mapped using separate protocols. The system utilizes a 3-part vest (CardioInsight Mapping Vest) with 252 unipolar electrodes, placed on the patient’s torso to record cardiac electrical activity. A 3D reconstruction is performed via a CT scan, segmenting cardiac structures at a spatial resolution of 6.8 mm. The software reconstructs electrograms, potential, and voltage maps across ~1400 nodes (Fig. 2). Activation and propagation maps are generated using the maximum negative deflection method (-dV/dT), and direction, phase, and composite maps are also available to illustrate impulse propagation, display rotors and focal activity. CardioInsight has been validated for localizing VPD and guiding ablation. A randomized controlled trial demonstrated that CardioInsight outperformed standard 12-lead ECG interpretation with 95.2% accuracy confirmed by invasive mapping [29]. A case series of patients with VPDs from the outflow tract showed 96% accuracy in identifying the chamber of origin [42]. However, another study comparing epicardial breakthrough and activation maps from CardioInsight with invasive contact mapping in epicardial procedures (notably Brugada syndrome and arrhythmogenic right ventricular cardiomyopathy cases) showed poor correlation, with an average breakthrough location difference of 52 mm [43]. Additionally, no anatomical correlation was found between lines of block, and mapping inaccuracies occurred in areas such as the right ventricular outflow tract during sinus rhythm.

Further limitations emerged when comparing low voltage area identification between CardioInsight and invasive electroanatomic mapping, showing overall only moderate correlation [44]. Since CardioInsight reconstructs the epicardial surface, it cannot provide information on septal low voltage areas. Two studies [18, 31] directly and simultaneously compared noninvasive mapping with CardioInsight and invasive electroanatomic mapping during different pacing configurations and VT. The first study quantitatively compared electrograms recorded simultaneously by both methods during various pacing modalities, revealing a spatial resolution of 13.2 mm at confirmed capture sites and moderate correlation for activation time maps (Pearson correlation coefficient 0.66), and even lower correlation for repolarization maps (0.55, probably due to lower T-wave amplitudes) [18]. These results were influenced by QRS duration (with poorer outcomes for narrower QRS complexes), anatomical misalignment, and filter settings, with up to a 25% variation for a 4 mm anatomical shift and reduced correlation with the default 50 Hz low-pass filter. The same co-registration protocol was later applied for mapping VT in patients with structural heart disease [31]. The median distance between the earliest activation site identified by CardioInsight and invasive mapping was 22.6 mm (better in non-ischemic vs ischemic cardiomyopathy patients 15.8 mm vs. 26.6 mm, *p* = 0.055, respectively). However, these results worsened when using the earliest negative voltage method instead of activation times. Despite the system’s inability to reconstruct septal structures, it mapped septal arrhythmias as accurately as those originating from other regions. Nonetheless, the system struggled to map the full macro-reentrant circuit and activation sequence of arrhythmias. Additionally, it could not reliably distinguish between epicardial and endocardial origins, as endocardial VTs did not exhibit rS complex electrograms at the origin site. These findings contrast with those of Duchateau [43], possibly due to higher system accuracy during paced rhythms or VT compared to sinus rhythm accompanied by manual signal editing to improve precision. Overall these experiences suggest CardioInsight’s resolution may be adequate to guide STAR but not a transcatheter ablation. This specific hypothesis is in fact under investigation in the CARA-VT RCT (NCT05047198), which compares STAR guided by CardioInsight with catheter ablation in patients with structural heart disease and recurrent VT.

CardioInsight has also been applied to analyze electrical activity in AF, using phase mapping to identify rotor and focal activity and distinguish active drivers from passive zones [45, 46]. Ablation at these sites has led to acute AF termination and favorable long-term outcomes [19, 20]. However, limitations exist: CardioInsight maps derive from overlaid structures and struggle to differentiate closely located regions like the non-coronary cusp and interatrial septum [47]. Additionally, phase mapping for rotor identification is prone to errors and false positives, particularly in cases where impulse propagation occurs in opposite directions parallel to a line of block [48, 49].

Finally, moving from activation to recovery, a ventricular electrical stability test, that calculates the relative change in electrogram (EGM) local activation times between a baseline and post-exertion phase using custom written software, was proposed to better understand and quantify effort-induced cardiac conduction heterogeneity among patients with Brugada Syndrome and idiopathic ventricular fibrillation (VF) [50]. This pilot study also underscores the fact that the CardioInsight vest can be used to record cardiac activity not only at rest but also during effort, paving new research possibilities in this field.

### 4.2 Amycard Mapping System

The Amycard system (Amycard 01C electrophysiology [EP] laboratory; EP Solutions SA, Yverdon-les-Bains, Switzerland), formerly EPCard IVM, enables the reconstruction and mapping of both epicardial and endocardial potentials across atrial and ventricular chambers. This system uses 224 unipolar body surface electrodes (28 stripes of 8 electrodes each) placed on the torso to record cardiac electrical activity. A recent study showed that reliable identification of VPDs or premature atrial complexes (PAC) can be achieved with as few as 74 electrodes. After electrode placement, a torso and cardiac CT or magnetic resonance imaging (MRI) scan is performed, followed by a 3D reconstruction of the torso and cardiac chamber geometries using proprietary software. The system solves the inverse problem using an extracellular potential model, reconstructing isopotential and isochronal maps on both surfaces, with over 2500 nodes. On top of activation maps, phase maps are also generated, identifying high-frequency activities and rotor dynamics. [51].

Current research with this system focuses on VT treatment and regional delay identification for optimizing cardiac resynchronization therapy (CRT). Results from the multicenter ICONIC-M (NCT05564793) study using Amycard for CRT optimizationare expected in 2026. A retrospective study showed that overlap between the latest electrical activation and left ventricle pacing site correlates with CRT response [52].

Validation studies typically involve pacing from various endocardial regions and comparing pacing sites with the first activation zone identified by the mapping system [53]. Amycard showed good agreement for pacing beats originating from the free ventricular walls, but showed lower performance for the septum, apical region, and outflow tract. Accuracy improved when the model was manually modified, such as by excluding the right ventricle for septal pacing sites [53].

In patients with cardiac implanted electronic devices, Amycard identified pacing site with a mean error of 6.8 millimeters, reduced to 5.5 millimeters during breath-holding. Similarly, in patients undergoing AF catheter ablation, it identified pacing sites in different atrial regions with an average error of ~7 millimeters (7.4 mm in the right atrium and 6.9 mm in the left) [54].

Amycard has proven effective in identifying VPD origins [55, 56, 57], and has guided ablation when arrhythmias were not present during the procedure [58]. A case series comparing Amycard system with the VIVO system showed good agreement in identifying VPD origins [51]. The system has also been validated for detecting monomorphic reentry VT in ischemic cardiomyopathy and for identifying rotor activity and multiple wavelengths in Brugada syndrome using phase mapping [59]. The system has also been employed to map rotors and focal activity in patients with AF, showing strong correlation with invasive endocardial mapping; however, rotor activity did not colocalize with regions of late gadolinium enhancement identified on MRI [60, 61]. Amycard phase mapping has also been utilized for mapping typical counterclockwise and clockwise atrial flutter, demonstrating good correlation with invasive data, although with some temporal delay compared to the invasive activation map [62, 63].

### 4.3 Acorys

The Acorys (Corify Care, Madrid, Spain) mapping system is distinct in its clinical development, as it primarily focuses on atrial mapping, particularly for AF treatment. Despite this focus, the system can map both atrial and ventricular chambers. It employs a proprietary vest with 128 electrodes to record cardiac electrical activity. A 3D camera is used to scan the patient’s torso and accurately determine the position of the electrodes. Unlike other systems, a CT or MRI scan of the cardiac structures is not strictly required [64], although integration with imaging data is possible. The system utilizes a proprietary AI software to generate chamber geometry and display mapping results [64]. The Acorys system reconstructs epicardial potentials and offers a variety of mapping options, including isopotential, activation, dominant frequency, and phase maps [65], with additional information on conduction velocity also available [66].

Most clinical studies using the Acorys system have focused on AF, specifically in identifying rotors and high-frequency drivers to better understand the arrhythmia mechanisms and potentially guide ablation [66, 67]. In addition to AF, the system has been applied in planning VT ablation procedures. Preliminary experiences have showed the role of the system in identifying VT isthmus during sinus rhythm by locating regions of slow conduction, an area of active ongoing research [68]. Another emerging application for Acorys is guiding the implantation of CRT devices or conduction system pacing, highlighting its expanding role in various cardiac interventions [69].

### 4.4 VIVO Mapping System

The View into Ventricular Onset (VIVO - Catheter Precision, Fort Mill, SC, USA) mapping system enables the reconstruction and mapping of epicardial, endocardial, and interpolated intramural potentials [70]. It is specifically designed for ventricular mapping, as it does not support mapping of atrial chambers. The system utilizes either a cardiac CT or MRI scan to delineate the patient’s 3D heart anatomy. A patient-specific heart model is reconstructed using proprietary software that integrates imaging data with a reference model [71]. A key feature is a 3D camera used to scan the patient’s torso, precisely localizing the 12 ECG leads and three proprietary reference patches. This is crucial because minor electrode position changes can significantly affect VPD localization accuracy [72]. The electrical cardiac source is modeled using an equivalent double-layer model that simulates the diffusion of depolarization in the myocardium. The resulting map directly displays isochrones of cardiac activation [70]. The model can theoretically also map the repolarization phase [73]. The origin of the VPD or VT exit site is identified by comparing the ECG-derived vector cardiogram with simulated ones for each node of the ventricular model at three key points: onset (30 ms after the QRS start), midpoint (0.5 × QRS duration), and end (0.8 × QRS duration) of depolarization [74, 75]. Manual identification of temporal markers, such as QRS onset and T-wave end, is required, possibly introducing some extra margin of error. In an ex-vivo experimental model, the system identified the pacing site with an average error of 18 mm [76].

**Fig. 3.**
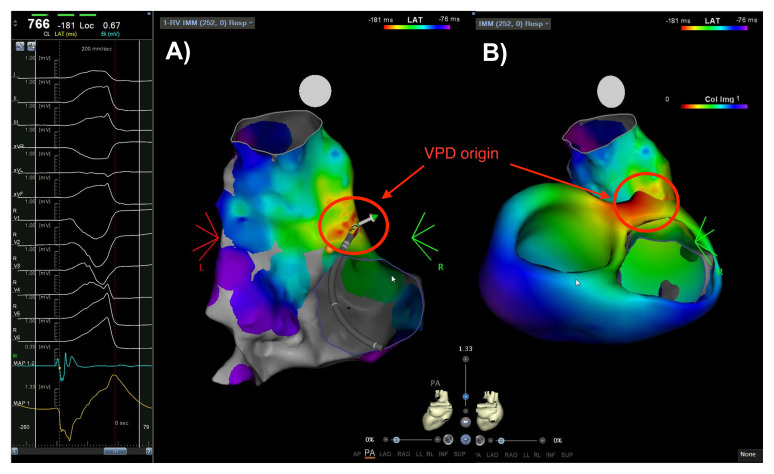
**Comparison between invasive mapping (A) and noninvasive mapping performed by the VIVO system (B), both in PA projection**. The maps show ventricular premature depolarization originating from the RVOT posterior wall (as highlighted by the red circles). The image shows a local activation map, with the earliest activation shown in red and the latest in blue or purple. In this case the correlation appears satisfactory despite in this region the noninvasive system is known to have a suboptimal resolution. Legend: PA, posteroanterior; RVOT, right ventricular outflow tract; VPD, ventricular premature depolarization.

VIVO has been validated for identifying the origin of VPDs or VT [70, 75]. In a case series of 20 patients (12 undergoing VPD ablation and 8 undergoing VT ablation), it correctly identified the focus of VPDs and VT in 85% and 88% of cases, respectively [74]. Another retrospective case series showed agreement with invasive mapping systems for VPD focus identification in 72% of cases (Fig. 3). This study emphasized that accurate origin prediction depends on the use of a patient-specific heart model and precise marker timing (e.g. a 5 ms shift in QRS onset or end can lead to significant errors), while patient positioning did not impact accuracy [77]. Case reports have demonstrated the feasibility of integrating the VIVO system into the ablation workflow and guiding the preferred access for mapping [78, 79]. An observational study reported that the VIVO system helped in reducing both procedure duration and radiation exposure [80]. Finally, in addition to the direct 12 leads resting ECG recording through the system software, that requires the target arrhythmia to be observed during the hospital monitoring, the possibility to upload data from a 12 leads Mortara (Hillrom) Holter ECG to the VIVO system, thereby expanding the observation time, was also reported [80, 81]. In these cases, the 3D photograph was taken using the VIVO camera while the traditional Holter leads were placed on the patient’s torso.

### 4.5 vMAP

vMAP (Vektor Medical, USA) uses an AI-based forward modeling approach to map cardiac electrical activity. vMAP compares a recorded 12-lead ECG with a library of over one million arrhythmia simulations generated using the Continuity platform [40, 82] (as of 2022). Patient-specific clinical characteristics, including body metrics and structural heart disease, are incorporated into the analysis [40]. The system requires a digitized 12-lead ECG and can map both atrial and ventricular chambers. The output is a heatmap displaying probability distribution across the cardiac surface, tailored to each arrhythmia type. For focal arrhythmias and pacing, it identifies the site of earliest activation; for macro-reentrant VT, it highlights the exit site and first myocardial activation; for orthodromic atrioventricular reentrant tachycardia (AVRT), it pinpoints the atrial insertion of the accessory pathway; and for AF and VF, it identifies reentrant areas potentially driving the arrhythmia [40]. vMAP is currently FDA-approved based on the pivotal VMAP study [40], where it showed 98.7% accuracy for chamber localization and 97.3% for segment-level identification. The median distance from invasive EP studies findings was 15 mm. For AF [83] and VF [83], the system identifies regions associated with local reentrant activity, as confirmed by intracavitary mapping with basket catheters. VPDs and PACs triggering VF and AF are mapped as focal arrhythmias. The registration study found no significant difference in accuracy between a generic anatomical model and a patient-specific 3D model. A retrospective study [84] showed that incorporating vMAP into clinical practice reduced both fluoroscopy and procedural times without compromising the efficacy or safety of the procedure. vMAP has been successfully used to guide cryoablation of a VPD focus during surgical unroofing of an anomalous right coronary artery [85]. Additionally, it has been employed in six patients undergoing STAR for VT, demonstrating feasibility in procedural planning [36], and in a case of perivalvular VT that was inaccessible to ablation due to the presence of mechanical mitral and aortic valves [33].

## 5. Current Limitations and Future Perspectives

Despite promising initial experiences, widespread clinical use of ECGi is limited by several aspects, as inaccuracies in epicardial breakthrough, low resolution of specific anatomical sites and lack of robust clinical evidence. Furthermore, geometric mismatches caused by cardiac and respiratory motion during image acquisition, minor electrode misplacement after the CT scan and during arrhythmia recordings, and challenges in manually segmenting anatomical structures can lead to anatomical misalignment and reduce reconstruction accuracy. The vMAP and Acorys systems, not requiring cardiac imaging and using non–patient-specific anatomical models, could potentially be less susceptible to anatomical misregistration.

Currently, noninvasive mapping is not primarily used for guiding transcatheter ablation, where invasive electroanatomical 3D systems remain more accurate for both substrate and activation mapping. However, ECGi is increasingly used to plan STAR procedures, helping identify target regions or areas of interest in very fast, poorly tolerated or unmappable VTs. In this scenario the integration of non-invasively collected ECGi maps integrated with anatomical details on the specific arrhythmic substrate represents, especially in those cases in which invasive mapping is not suitable, a real advantage. In addition, a growing area of research is the application of ECGi to study not only depolarization (activation), but also repolarization dynamics (at rest but also during effort), in order to improve arrhythmic risk stratification in several cardiac disorders, spanning channelopathies (Long QT syndrome [86] and Brugada syndrome [87]) and arrhythmogenic cardiomyopathy [88] to idiopathic ventricular fibrillation [50, 89].

A recent analysis using the CADIS-ECGI system showed significant differences between LQTS patients and controls in both activation and recovery sequences. These analyses may provide a visual and quantitative measure of repolarization dispersion and potentially add to arrhythmic risk stratification [90].

Finally, ongoing studies are further investigating other potential clinical scenarios: the GUIDE study (NCT06509763) will test the ability to assess resynchronization in patients undergoing left bundle branch pacing; the BREACH-ECGI (NCT04548804) aims to specifically shed light on arrhythmic risk stratification for patients at increased risk of ventricular arrhythmias in different settings (from channelopathies to structural heart disorders).

## 6. Conclusion

Noninvasive mapping using ECGi systems for atrial and ventricular arrhythmias is feasible and should be considered in specific clinical settings, such as STAR procedures planning, particularly for patients without an available invasive electroanatomic map. Several commercial solutions exist, but there is currently no clear evidence favoring one system over. The ENCORE-VT trial further suggests that noncommercial, research-oriented solutions (CADIS-ECGI system [1, 25, 26]) can also be effective.

If the CARA-VT trial yields positive results, the CardioInsight system would be the only ECGi solution with randomized evidence supporting its use. The VIVO mapping system offers the theoretical advantage of reconstructing both endocardial and epicardial surfaces with higher anatomical resolution for septal arrhythmias compared to CardioInsight, although it lacks other additional features. The Amycard system also supports both endocardial and epicardial reconstructions, retaining some CardioInsight’s features, but current clinical studies primarily focus on its role in cardiac resynchronization therapy. Similarly, while the Acorys system was originally designed for AF studies, promising data are now emerging regarding its application in managing ventricular arrhythmias. The vMAP and Acorys systems offer the benefit of not requiring cardiac imaging, making them more accessible for planning catheter ablation procedures. However, this may be less advantageous in the STAR workflow, where more detailed anatomical information is often necessary.

Given these evidence, despite presenting several potential advantages, invasive electroanatomic 3D mapping remains the gold standard and should be preferred whenever possible, as each noninvasive system has limitations that require further research.
